# Financial Capacity Assessment in Female Euthymic Bipolar Patients: Catching Up on a Long Neglected Vulnerable Group

**DOI:** 10.3390/healthcare13131607

**Published:** 2025-07-04

**Authors:** Vaitsa Giannouli

**Affiliations:** School of Social Sciences, Hellenic Open University, 263 31 Patras, Greece; giannouliv@hotmail.com

**Keywords:** financial capacity, financial decision-making, financial exploitation, self-estimations, euthymic, bipolar disorder

## Abstract

**Background**: Patients with bipolar disorder (BD) face many challenges as many basic cognitive and non-cognitive domains can be affected by their disease. Financial capacity requires complex cognitive functioning and is little investigated in BD, especially in the Greek cultural context. **Objectives**: This study, for the first time, is focusing on whether financial capacity shows deficits in female euthymic BD patients compared to controls and what the self-estimations of the patients for their performance are. **Materials and Methods**: Patients and a sample of one-to-one matched healthy controls were examined with a detailed neuropsychological battery and the Legal Capacity for Property Law Transactions Assessment Scale (LCPLTAS). Before their neuropsychological assessment, participants responded to a single-item five-point Likert scale about their financial capacity. **Results**: Findings extend earlier work in other groups of older patients and indicate that euthymic BD patients’ performance is lower than that of the control group in various subdomains and total score of LCPLTAS (*p* < 0.001), resembling the performance of patients with a diagnosis of Mild Cognitive Impairment (MCI). However, euthymic BD patients are not aware of their cognitive deficits compared to healthy controls and overestimate their financial capacities as they have more positive estimations regarding their financial capacity than controls (χ^2^(1) = 8.315, *p* = 0.004) despite their lower real performance. In addition, from a number of classic neuropsychological tests administered, only Trail Making Part B correlates with LCPLTAS scores for the group of euthymic BD patients (rho = −0.561, *p* = 0.005). **Conclusions**: The results support that special care must be provided for euthymic BD individuals, so we can prevent financial exploitation.

## 1. Introduction

Marked cognitive deficits have been found by several studies for euthymic bipolar disorder (BD) patients that is for BD patients who experience a state of normal or stable mood, not characterized by mania or depression [1]. These deficits focus on cognitive domains such as inhibitory control and selective attention as well as verbal and visual memory [2]. The impaired neuropsychological performance is associated with factors such as the duration of illness, the total number of episodes per lifetime, and the previous episodes with psychotic features [2]. Despite this support for impairment in the cognition of euthymic BD patients, so far we know little about more complex cognitive capacities, such as financial capacity. Although numerous studies support declining and impaired financial capacity in amnestic Mild Cognitive Impairment (aMCI) and aMCI with concurrent depressive symptomatology (aMCI-D) [3,4], we still have no empirical data for the role of mood on objectivelymeasured financial capacity in BD patients. Financial capacity is considered to be an instrumental activity of daily living playing the role of the single best predictor of capacity for independent living as studies show inolder adults [3]. Although it is complex, as it comprises a variety of activities and specific skills, including performance skills (such as arithmetic counting coins/currency, paying bills, etc.) and judgment–decision-making skills [3], no systematic research on BD patients exists so far.

Data (not specifically focusing on euthymic BD) support that poor mental health can lead to financial behaviors such as compulsive buying, whereas worry about finances increases anxiety and stress, with a vicious cycle for anxiety [5]. A similar study shows that BD patients have fewer financial management skills and higher levels of impulsivity than controls, while increased impulsivity is associated with poorer personal financial management [6]. In BD, during manic or hypomanic phases, patients may exhibit prodigal behavior, spend impulsively large amounts of money, and possibly engage in risky financial activities which are usually measured with self-report questionnaires or third-party estimations and not measured with relevant objective tests/tasks [7,8]. These episodes are typically marked by poor disease awareness and lack of insight, but at the same time this diminished awareness has a great impact on the quality of life specifically of the euthymic BD patients [9], therefore rendering these sources of information partly unreliable for clinicians and making increasingly important the need for objective testing [10,11].

Although there is scarce research so far, following the same assessment protocols of financial capacity in patients coming from different countries–cultures, it has been supported that the role of the cultural context is of extreme importance in such measurements as certain dimensions of culture are strongly correlated with financial capabilities in healthy participants. Positive relationships have been found between financial capability and three cultural factors—Individualism, Long-Term Orientation, and Indulgence—while Uncertainty Avoidance has been found to negatively associate with financial capabilities [12]. Despite the epidemiological data gaps, in Greece, a prevalence for BD I and II is estimated approximately as 1%, although no officially published statistics are provided for the common mental disorders due the lack of representative samples of the population from both mainland and insular regions of the country [13].

Thus, the main objective of this study is to examine for the first time the financial capacity performance in the neglected group of euthymic BD patients and compare their performance to healthy adults with the exact same demographic characteristics. In addition to that, this study aims to focus solely on a little investigated cultural background by examining not only Greek euthymic BD patients’ real-life performance, but also their self-reports regarding their financial capacity.

## 2. Methods

### 2.1. Sample and Data Collection

In total, 28 adults (all women; age of 31–60 years) from Greece took part in the study. Only female participants were selected based on the fact that in Greek households, women manage finances, so the abovementioned question is of practical importance. A total of 14 females with a BD diagnosis (M_age_ = 48.50, SD = 7.01; M_education_ = 15.21, SD = 1.76), who were in euthymic state for at least 3 months, with a minimum 2 years of illness duration and 14healthy controls (M_age_ = 46.42, SD = 7.76; M_education_ = 14.28, SD = 2.99), participated in the study. The small size is due to the fact that this study aims to examine a specific subgroup of BD patients, namely female euthymic BD patients with a detailed assessment protocol. This choice may have limitations in the generalizability and the statistical power to detect meaningful differences in this study.

All participants completed the Hamilton Depression Rating Scale (HAM-D) (inclusion score < 6) [14] and the Young Mania Rating Scale (YMRS) (inclusion score < 7) [15] so the euthymia state was assessed as the lack of signs of depression and/or mania. The absence of depressed or elevated mood was also confirmed by the doctors taking care of the patients before the neuropsychological testing took place. Snowball sampling was followed for the BD group and the researcher used referrals from initial participants who were members in patient support groups (online and offline) in order to generate additional participants, while the control group of the community-dwelling individuals were approached following purposeful sampling that is creating a group of participants that had similar characteristics with the patient group. Patients and controls were not recruited from the same clinical setting.BD patients had a psychiatric evaluation from different private and public clinics and psychiatrists across the country following the DSM-5 diagnostic criteria.

Healthy controls had no depressive and/or manic symptomatology, were not under antidepressant or other treatment at the time of the examination, and were free of a diagnosis related to cognitive decline. Immediately after completing the data collection for the patient group, healthy participants were recruited and assessed. Their gender, age, and education characteristics were matched on a one-to-one basis with the characteristics of the patient group. Therefore, healthy participants were matched with the euthymic BD group regarding age [U = 114.500, *p* = 0.461] and years of education [U = 123.500, *p* = 0.196] in order to eliminate a possible influence of the abovementioned variables as confounders. Explicit exclusion criteria for all participants were known history of stroke, traumatic brain injury and related neurosurgical interventions, a history of substance abuse, concomitant serious medical illness (such as significant visual and/or auditory impairment not corrected sufficiently by visual/auditory aids), and a history of another neurologic or psychiatric disorder.

### 2.2. Neuropsychological Tests and Assessment

Several cognitive domains were examined with a number of standardized neuropsychological tests:

- The Trail Making Test (Parts A and B; TMT-A and TMT-B) which examines attention and working memory. In Part A, participants were required to connect numbered circles in ascending order and numbers and letters of the alphabet in ascending order for the Part B. For both tests the time to completion in seconds was measured.
- The Digit Span forward subtest of the Wechsler Adult Intelligence Scale III (which measures immediate attention) as well as the Digit Span (WAIS-III) greatest span backwards test which measures working memory capacity.
- The verbal fluency/phonemic fluency test that is the total number of words generated in one phonological category, namely words starting with the Greek letter Chi.
- The verbal memory test which examines memory and learning of a 10-word list repeated over four trials. The outcome included in the analyses is the number of words on immediate and delayed recall conditions of the test,
- The Rey–Osterrieth Figure Test (RCFT) which measures visuospatial abilities and memory through three conditions: copy condition, immediate and delayed recall scores.

Financial capacity was assessed with the Greek full version of the Legal Capacity for Property Law Transactions Assessment Scale (LCPLTAS) [16]. The LCPLTAS extended form consists of seven main domains: (1) basic monetary skills, (2) cash transactions, (3) bank statement management, (4) bill payment, (5) financial conceptual knowledge, (6) financial decision-making, and (7) knowledge of personal assets [16]. In this sample, the LCPLTAS demonstrated a satisfactory internal validity (Cronbach’s alpha was 0.760). Euthymic BD patients and controls were also asked (but not obliged) to respond to a single 5-point Likert scale/self-report question before testing with LCPLTAS: “How do you rate your financial abilities?” (answers: 1 = very poor, 2 = poor, 3 = ok, 4 = good, 5 = very good).

### 2.3. Ethical Issues

Informed consent was obtained for data collection from all the participants and prior to their admission to the study. The study was approved by the Ethics Committee of the Aristotle University of Thessaloniki (as part of a larger study with diverse populations of adult participants and diagnoses [16]), and it was performed according to the declaration of Helsinki.

### 2.4. Data Analysis

The Statistical Package for Social Sciences (SPSS) version 28.0 (IBM Corp, Armonk, NY, USA) was used. Means and standard deviations were used for demographic variables and in order to explore the nature of neuropsychological functioning. Spearman’s correlations were used to study the relationship of objective financial capacity performance (LCPTLAS) with cognitive functioning as measured by neuropsychological tests and self-estimations of financial capacity as measured with the single self-report question. Mann–Whitney U tests were used as a non-parametric test in order to compare the cognitive test scores of the two groups. A chi square test was used to estimate differences in proportions regarding the reports in the Likert scale and the group membership (patient/nonpatient). Only raw scores were calculated for the LCPTLAS and neuropsychological performance in the other administered tests. Probability values < 0.05 (two-tailed) were considered statistically significant.

## 3. Results

A number of statistically significant differences were found for the neuropsychological performance in classic tests (see Table 1). Although in all tests the healthy controls had better scores than euthymic BD, statistically significant differences were found for the immediate memory of story, Trail Making Parts A and B, and Digit Span Backward.

It is of interest that the performance of healthy controls was superior to that of euthymic BD individuals with statistically significant differences not only in the total score of LCPLTAS, but also in all its subscales (Table 2). Furthermore, the effect size for the for Mann–Whitney U test for the total score of LCPLTAS, which is the rank-biserial correlation r, shows a very large effect size (equal to −1), with the group of healthy controls performing extremely higher than the euthymic BD group and the magnitude of the negative r reflecting the strength of this negative relationship. Although higher objective financial performance was observed in the healthy controls, euthymic BD individuals had higher self-reports regarding their financial capacities (M = 4.76, SD = 0.43) compared to healthy controls (M = 4.21, SD = 0.42; U = 141.50, *p* = 0.005). Additionally, chi square analysis revealed a statistically significant association between group membership (being an euthymic BD patient) and higher self-reports for financial capacity (χ^2^(1) = 8.315, *p* = 0.004) (see Table 3).

When Spearman’s correlations were calculated for the patient group, only one strong negative correlation was found between the Trail Making Part B and the total score of LCPTLAS (rho = −0.561, *p* = 0.005), while all other neuropsychological test scores such as the Trail Making Part A (rho = −0.004, *p* = 0.991), the Digit Span Forward (rho = 0.088, *p* = 0.786), the Digit Span Backward (rho = 0.034, *p* = 0.915), the verbal fluency test (rho = 0.554, *p* = 0.062), the word list learning (rho = 0.051, *p* = 0.875), the RCFT copy (rho = 0.009, *p* = 0.978), the RCFT immediate recall (rho = 0.107, *p* = 0.740), and the RCFT delayed recall (rho = 0.060, *p* = 0.862) did not correlate in a statistically significant way with the total score of LCPTLAS.

## 4. Discussion

These preliminary findings provide support for difficulties regarding financial capacity in female euthymic BD patients that are highly correlated to executive functioning (as measured by Trail Making Part B). Performance in euthymic BD resembles more with the scores recorded in Mild Cognitive Impairment (MCI) (as indicated by a previous study presenting the norms of this tool for the Greek population, M_LCPTLAS_ = 182.42, SD = 27.66) [16]. This is considered as a reaffirmation that deficits in cognition may be present even in the euthymic BD phase given that other studies show that altered brain functioning in several important brain regions such as the postcentral gyrus, middle frontal gyrus, medial frontal gyrus, and superior frontal gyrus [17] can also lead to cognitive impairment in BD, something that may contribute to poor functional outcomes such as diminished financial decision-making skills.

This is the first study to show that euthymic BD status does have a differentiating power on financial capacity performance. Although there is a paradox that appears as lower objective performance accompanied by simultaneous higher self-estimations of performance for financial tasks in this group. This overestimation has also been reported for patients with frontotemporal dementia [18] as well as for MCI patients [19] and patients with dementia with Lewy Bodies [20].

These findings present the detrimental influence of BD as a diagnosis on financial capacity and is inline with previous research which has established that deficiencies in cognitive functions may persist after clinical recovery or in remitted BD patients and may prevent patients from attaining an optimal adaptation in their daily lives [21,22,23,24]. What is of interest is that despite the low objective performance in financial tasks, the self-reports of euthymic BD patients regarding these very tasks were higher than the controls, thus showing not only overestimations, but actually lack of self-awareness that may lead them to situations of financial exploitation by third parties.

The findings of deficits in financial performance could be explained by previous research which supports that the amygdala as a brain structure influences financial skills in older MCI patients [25], something that is also found in a different research that supports that the amygdala functional activity and its connectivity to other brain regions remains altered in euthymic BD patients [26]. In addition, the domains of the cognitive deficits found in euthymic BD patients in this sample (but also reported in a substantial proportion of such patients in large-scale studies from abroad) [27,28,29], namely reduced performance on tasks measuring verbal memory, psychomotor speed, and visuospatial ability and especially cognitive flexibility/executive functions [2,27] are all potentially involved in the impairment of financial capacity also in MCI. Although the persistent deficits in some cognitive domains appear similarly affected in euthymic BD patients and MCI patients, this does not imply that patients with BD experience comparable levels of social functioning with MCI patients. In BD, quality of life and functional outcomes are significantly influenced by key clinical variables, such as illness course, number of hospitalizations, and frequency and severity of manic/hypomanic and depressive episodes [30], and thus it should be emphasized that BD and MCI are distinct conditions, even if they share certain cognitive correlates related to financial capacity.

One last point to be highlighted here is that although a previous study supports that financial domain is the least affected compared to others (such as occupational domain and interpersonal) in euthymic BD based on a succinct examination of this domain based on the Functioning Assessment Short Test (FAST) scale [31], this is not the case when more detailed assessment of financial capacity and financial decision-making is employed.

These findings are preliminary, but can inform screening of BD patients in Greece given that financial problems and relevant decision-making can be easily tested with the proposed test (LCPTLAS) as it has already been used in assessment protocols of patients with dementia in Greece. Also this study supports that intervention strategies in clinical practice for BD patients must be tailored by the specific financial capacity deficits they show in relevant subdomains of LCPTLAS and the only way to achieve this is through individualized and standardized testing of these problems. Although this research did not focus on moderator/mediator variables such as personality or other psychological variables, such as dependency and achievement cognitions and/or mindfulness were examined in this study [32], future research and practice should be based on such person-centered approaches that include more possible influencing factors [33].

## 5. Limitations and Implications

Despite the significant contribution of the current results, a number of limitations should also be outlined. The most important limitation is that the sample size was relatively small and not representative of euthymic BD patients. More specifically, male participants were excluded as a homogeneous sample regarding the demographic characteristics was desired. This methodological approach makes the findings more focused on women’s financial performance and decision-making, but at the same time disregards the differential influence of gender-based roles in household financial management and the established clinical differences in BD across genders, as well as the impact of financial capacity on quality of life, which is significant regardless of gender-assigned-at-birth.

Furthermore, relevant variables such as the occupational status which relates to cognitive and functional status (with employed euthymic BD patients presenting greater functioning and interpersonal relationships, as well as better verbal learning performance and speed of processing) [34] could not be included in the current analyses as all participants worked full or part-time, so caution is needed in the extrapolation of these results to the general population or people with BD [35].

Despite the fact that this is one of the few studies focusing on this specific area of functioning, the respective implications for practice, research, and education are numerous. For example, financial capacity and financial decision-making seem to be domains which are still affected even in the euthymic BD, therefore making it necessary to include them in all assessments of financial autonomy. A way to approach this in future assessments could be through the introduction not only of tests such as the LCPLTAS, but also by examining relevant psychological variables such as impulsivity, inhibitory control, and delay of gratification [36]. Based on this, healthcare professionals must be educated regarding these potential problems in order to examine and prevent financial exploitation due to these deficits in individuals with euthymic BD.

## 6. Conclusions

According to this study financial capacity performance in euthymic BD is lower from the healthy controls’ mean performance, something that may render this population vulnerable to financial exploitation. Moreover, the utility of this neuropsychological finding should be examined in larger samples in future longitudinal studies, so targeted interventions can be designed for these patients.

## Figures and Tables

**Table 1:** Performance in neuropsychological tests for the two groups.

Neuropsychological Tests	Group	Mean Score	SD	*p*
RCFT copy	Euthymic BD	31.71	5.18	0.090
	Healthy	34.77	1.09	
RCFT immediate recall	Euthymic BD	18.32	3.95	0.782
	Healthy	19.21	9.34	
Word list learning	Euthymic BD	30.42	5.15	0.062
	Healthy	33.92	3.40	
Immediate memory of story	Euthymic BD	21.07	4.85	0.001
	Healthy	25.57	2.13	
Trail Making Part A (in seconds)	Euthymic BD	51.14	14.70	0.016
	Healthy	37.17	8.63	
Trail Making Part B (in seconds)	Euthymic BD	84.15	31.21	0.003
	Healthy	55.57	11.14	
Delayed memory of words	Euthymic BD	7.21	1.88	0.794
	Healthy	7.64	1.08	
Word recognition	Euthymic BD	19.50	0.85	0.525
	Healthy	19.69	0.63	
Delayed memory of story	Euthymic BD	11.64	3.97	0.067
	Healthy	13.85	1.74	
RCFT delayed recall	Euthymic BD	12.23	7.82	0.348
	Healthy	14.55	6.73	
Digit span backward	Euthymic BD	5.00	1.79	0.037
	Healthy	6.53	1.12	
Digit span forward	Euthymic BD	6.85	2.07	0.803
	Healthy	6.53	1.76	
Phonological fluency (chi total)	Euthymic BD	8.50	3.41	0.678
	Healthy	9.50	4.46	

**Table 2:** Financial capacity performance in euthymic BD and healthy controls as measured by LCPLTAS and 7 sub-scales consisting LCPLTAS.

Measures of Financial Capacity	Group	Mean	SD	*p*
Total LCPLTAS	Euthymic BD	177.50	25.62	0.000
	Healthy	211.00	1.35	
Subscales of LCPLTAS				
Basic monetary skills	Euthymic BD	11.91	2.31	0.002
	Healthy	14.00	0.00	
Cash transactions	Euthymic BD	6.00	1.20	0.000
	Healthy	8.00	0.00	
Bank statement management	Euthymic BD	6.08	0.90	0.000
	Healthy	8.00	0.00	
Bill payment	Euthymic BD	6.66	1.37	0.005
	Healthy	7.85	0.36	
Financial conceptual knowledge	Euthymic BD	29.08	2.42	0.000
	Healthy	31.92	0.26	
Financial decision-making	Euthymic BD	92.25	16.19	0.000
	Healthy	113.50	0.85	
Knowledge of personal assets	Euthymic BD	25.50	3.45	0.026
	Healthy	27.71	0.61	

**Table 3:** Contingency table for financial capacity self-reports.

Diagnostic Group	Self-Reports in Likert Scale		Total
	4	5	
Euthymic BD	3	10	13
Healthy	11	3	14
Total	14	13	27

## Data Availability

The data presented in this study are available on request from the corresponding author. The data are not publicly available due to privacy issues.
